# Charakteristika von Antragstellenden bei erstmaliger Feststellung einer Pflegebedürftigkeit – eine bundesweite Analyse von Pflegebegutachtungen von AOK-Versicherten ab 60 Jahren

**DOI:** 10.1007/s00391-024-02344-x

**Published:** 2024-08-27

**Authors:** Christine Haeger, Till Baldenius, Susanne Schnitzer, Kathrin Jürchott, Adelheid Kuhlmey, Stefan Blüher, Antje Schwinger

**Affiliations:** 1 Institut für Medizinische Soziologie und Rehabilitationswissenschaft, Charité – Universitätsmedizin Berlin, corporate member of Freie Universität and Humboldt Universität zu Berlin, Charitéplatz 1, 10117 Berlin, Deutschland; 2 Wissenschaftliches Institut der AOK (WIdO), Rosenthaler Straße 31, 10178 Berlin, Deutschland

**Keywords:** Medizinischer Dienst, Regionale Unterschiede, Soziale Determinanten, ICD-10-Diagnosen, Pflegegrad, Medical Service, Regional differences, Social determinants, ICD-10 diagnoses, Care level

## Abstract

**Hintergrund:**

Die steigende Zahl Pflegebedürftiger erfordert präventive Ansätze, um Pflegebedürftigkeit zu verhindern oder Selbstständigkeitsverluste zu reduzieren. Die Pflegebegutachtung kann wertvolle Hinweise dazu liefern.

**Ziel der Arbeit:**

Das Ziel dieses Beitrags ist es, soziodemografische Merkmale von Personen mit festgestellter Pflegebedürftigkeit zu beschreiben, sowie eine differenzierte Betrachtung der erstgenannten pflegebegründenden Diagnosen nach Alter, Geschlecht, Pflegegrad (PG) und Bundesland.

**Material und Methoden:**

Als Datengrundlage dient ein bundesweiter Datensatz der Pflegebegutachtungen des Medizinischen Dienstes (MD) von AOK-Versicherten ab 60 Jahren, die im Jahr 2021 erstmalig einen PG erhielten. Pflegerelevante Informationen werden deskriptiv ausgewertet.

**Ergebnisse:**

339.486 Personen mit einem Durchschnittsalter von 79,6 Jahren (± 8,4), davon 59,0 % Frauen, wurden analysiert. Etwa die Hälfte erhielt den PG 2, 32,4 % den PG 1. PG 3–5 wurden seltener begutachtet (16,2 % vs. 4,8 % vs. 1,7 %). Alleinlebende waren öfter in niedrigeren PG vertreten, und es zeigte sich ein überproportionaler Anteil aus den neuen Bundesländern. Die Top‑3 der erstgenannten pflegebegründenden Diagnosen waren Senilität (R54), Polyarthrose (M15) und Demenz (F03), wobei, stratifiziert nach Bundesländern, große Unterschiede zu erkennen sind (ICD-10 R-Kapitel 0,8 % Berlin und Brandenburg vs. 37,9 % Sachsen; M‑Kapitel: 13,6 % Bayern und Hamburg vs. 39,9 % Mecklenburg-Vorpommern).

**Diskussion:**

Soziale Determinanten wie Alter, Geschlecht, Alleinleben und Region können bei der Einstufung in einen PG eine Rolle spielen. Auffällig sind große Unterschiede bei den erstgenannten pflegebegründenden Diagnosen zwischen den Bundesländern, was in zukünftiger Forschung genauer untersucht werden sollte.

## Abstract (en)

**Background:**

The increasing number of care-dependent individuals requires approaches to prevent care dependency or reduce the loss of independence. Long-term care assessments can provide valuable insights into this.

**Objective:**

The aim of this article is to describe initial applicants with an identified need for long-term care as well as to provide a differentiated analysis of care-related diagnoses by age, gender, care level and federal state.

**Material and methods:**

The nationwide database consists of long-term care assessments conducted by the Medical Service (MD) of individuals insured with the AOK aged 60 years and above who received a care level (PG) for the first time in 2021. Information relevant to long-term care was analyzed descriptively.

**Results:**

In this study 339,486 individuals with an average age of 79.6 years (±8.4 years) and a female proportion of 59.0% were analyzed. Approximately one half received care level 2 and 32.4% received care level 1. Care levels 3–5 were assessed less frequently (16.2% vs. 4.8% vs. 1.7%, respectively). Individuals living alone were represented more strongly in lower care levels, while individuals not living alone had a higher proportion in care levels 3–5. The most frequent care-relevant diagnoses were senility (R54), polyarthritis (M15) and dementia (F03) with significant differences observed between federal states (ICD-10 R chapter: 0.8% Berlin and Brandenburg vs. 37.9% Saxony; M chapter: 13.6% Bavaria and Hamburg vs. 39.9% Mecklenburg-Western Pomerania).

**Conclusion:**

Social determinants, such as age, gender, living alone, and region can play a role in the classification into a care level. Significant differences in care-related diagnoses between federal states warrant further investigation in future research.

## Hintergrund und Fragestellung

In Deutschland erleben wir derzeit eine Alterung der Bevölkerung aufgrund gestiegener Lebenserwartung und rückläufiger Geburtenraten [1]. Prognosen zufolge wird im Jahr 2060 etwa ein Viertel der deutschen Bevölkerung über 65 Jahre alt sein [2], mit Folgen für das Gesundheitssystem, insbesondere durch eine Zunahme pflegebedürftiger Personen [3]. Angesichts dieser Entwicklung müssen die Versorgungsstrukturen weiterentwickelt und Strategien zur Reduzierung der Nachfrage nach Pflegeleistungen entwickelt werden. Präventive und rehabilitative Maßnahmen sind entscheidend, um Pflegebedürftigkeit zu verhindern, zu mildern oder zu verzögern. Diese Maßnahmen sollten zielgruppenspezifisch sein, um gesundheitliche Ungleichheiten im hohen Alter zu verringern. Dafür ist es notwendig, Pflegebedürftigkeit unter Berücksichtigung sozialer Determinanten wie Alter, Geschlecht, Region oder Wohnsituation zu analysieren [4]. Pflegebedürftigkeit, ein Zustand hoher Vulnerabilität in physischen, psychischen und sozialen Dimensionen, wird sozialversicherungsrechtlich durch den Medizinischen Dienst (MD) gemäß SGB XI erfasst [5]. Seit der Neuausrichtung des Pflegebedürftigkeitsbegriffes 2017 wurden die MD-Daten in 2 Veröffentlichungen aus dem Modellprogramm zur Weiterentwicklung der Pflegeversicherung [6, 7] und 2 weiteren Studien [8, 9] ausgewertet. Diese konzentrierten sich auf die Verteilung über die Pflegegrade (PG), die Charakteristika der Antragstellenden und die Potenziale aus den Empfehlungen. Es ist jedoch zu beachten, dass diese Arbeiten regionale Analysen darstellen (Bayern [8] und Berlin/Brandenburg [7, 9]) bzw. einen kurzen Zeitraum [6] umfassen. Eine deutschlandweite Analyse zur jährlichen Inzidenz von Pflegebedürftigkeit fehlt bislang, da die Darstellungen des Statistischen Bundesamtes nur Pflegeprävalenzen abbilden [10]. Zudem fehlen in der Pflegestatistik Angaben zu pflegebegründenden Diagnosen und sozialen Merkmalen wie der Wohnsituation. Diese Forschungslücke wird adressiert mit dem vorliegenden Beitrag, der folgende Fragestellungen untersucht: 1) Wie viele AOK-Versicherte ab 60 Jahren, die erstmalig 2021 einen Antrag auf Pflegebedürftigkeit stellen, werden pflegebedürftig (1-Jahres-Inzidenz)? 2) Anhand welcher soziodemografischen Merkmale und pflegebegründenden Diagnosen lässt sich diese Population beschreiben? 3) Gibt es regionale Unterschiede in den pflegebegründenden Diagnosen zwischen den Bundesländern?

## Studiendesign und Untersuchungsmethoden

Im Rahmen des Projektes „WEGE – Analysen von Versorgungsverläufen bei älteren AOK-Versicherten im Vorfeld einer Pflegebedürftigkeit“, durchgeführt von der Charité – Universitätsmedizin Berlin in Kooperation mit dem Wissenschaftlichen Institut der AOK (WIdO), werden präventive Potenziale der Pflegebedürftigkeit untersucht. Datengrundlage des vorliegenden Beitrags sind bundesweite anonymisierte MD-Begutachtungsdaten aller AOK-versicherten Personen ab 60 Jahren, die im Jahr 2021 erstmalig einen erfolgreichen Antrag auf einen PG gestellt haben (*n* = 339.486).

Folgende Merkmale konnten für die Analysen herangezogen werden: Alter, Geschlecht, erste pflegebegründende Diagnose (ICD-10, 3‑Steller), PG (1–5), regionale Zugehörigkeit (Ost/West; städtisch, Agglomeration, ländlich), Wohnsituation (alleinlebend) sowie Art der Begutachtung (persönlich, per Aktenlage). Die Begutachtenden haben die Möglichkeit, bis zu 2 im Wesentlichen pflegebegründende Diagnosen – basierend auf vorliegenden ärztlichen Befunden und/oder Selbsteinschätzung – in die Gutachten aufzunehmen [5]. In die nachfolgende Analyse geht die erstgenannte Diagnose ein; im Folgenden als MD-Diagnose bezeichnet. Die regionale Zugehörigkeit wurde anhand des siedlungsstrukturellen Regionstypen des Bundesinstituts für Bau‑, Stadt- und Raumforschung (BBSR) angegeben. Hier wird in städtische Regionen, Regionen mit Verstädterungsansätzen (Agglomeration) und ländliche Regionen unterschieden [11]. Die Codierung der MD-Diagnosen wurde zusätzlich nach Bundesländern spezifisch ausgewertet.

## Ergebnisse

Im Jahr 2021 erhielten 339.486 AOK-Versicherte ab 60 Jahren erstmals einen PG. Das Durchschnittsalter lag bei 79,6 Jahren (±8,4). Die soziodemografischen und pflegebezogenen Charakteristika der Stichprobe sind in Tab. 1 dargestellt. Die meisten Fälle entfielen auf PG 1 und 2 (32,4 % bzw. 44,9 %). Mehr Frauen als Männer erhielten eine Pflegeeinstufung (59,0 % vs. 41,0 %), wobei Frauen in PG 1 und 2 und Männer in den höheren PG 3–5 dominierten. Nichtalleinlebende Personen erhielten etwas häufiger einen PG als Alleinlebende (49,6 % vs. 40,4 %).

**Tab. 1:** Charakteristika der AOK-Versicherten ab 60 Jahren die im Jahr 2021 erstmalig einen Pflegegrad (PG) anerkannt bekommen haben, *n* = 339.486

	Gesamt	PG 1	PG 2	PG 3	PG 4	PG 5
	*n*	*n* (%)	*n* (%)	*n* (%)	*n* (%)	*n* (%)
	339.486	109.995 (32,4)	152.504 (44,9)	55.034 (16,2)	16.320 (4,8)	5633 (1,7)
	% von Gesamt	% von PG 1	% von PG 2	% von PG 3	% von PG 4	% von PG 5
*Altersgruppen*^1^						
60–69	15,2	16,0	13,8	15,6	18,9	21,8
70–79	25,6	26,7	24,6	25,4	27,8	29,3
80–89	49,9	50,4	51,3	48,0	42,7	39,8
90+	9,3	6,8	10,3	11,1	10,6	9,1
*Geschlecht* (weiblich)	59,0	66,2	59,3	49,5	45,4	43,5
*Region*						
Ost^2^	28,4	27,1	28,9	29,2	28,6	34,8
West	71,6	72,9	71,1	70,8	71,4	65,2
*Verdichtungsraum*						
Städtisch	38,9	37,7	39,0	40,8	39,6	36,8
Agglomeration	34,1	34,7	33,7	33,5	34,9	35,7
Ländlich	27,1	27,6	27,3	25,7	25,5	27,5
*Alleinlebend* (ja)^3^	40,4	53,6	41,7	23,5	8,1	5,2
*Art der Begutachtung*						
Aktenlage aus sonstigen Gründen	61,2	62,3	63,8	62,4	43,9	38,3
Persönliche Befunderhebung	29,8	35,2	30,7	23,7	12,6	9,0
Aktenlage, weil Antragsteller verstorben	3,9	0,1	0,8	8,4	30,7	39,2
Aktenlage, weil persönliche Befunderhebung nicht zumutbar	2,4	0,6	1,4	5,0	12,1	12,6
Keine Angabe	2,2	1,7	3,4	0,5	0,7	0,9

^1^Der Gesamtaltersdurchschnitt liegt bei 79,6 (SD: 8,4)

^2^Hierunter zählen: Berlin, Brandenburg, Mecklenburg-Vorpommern, Sachsen, Sachsen-Anhalt, Thüringen

^3^Andere Wohnformen sind: vollstationäre Pflegeinrichtung, Einrichtung für Menschen mit Behinderungen, ambulante Wohnsituation mit anderen, ambulant betreute Wohngruppe. Für die Analyse stand nur das Merkmal „alleinlebend“ zur Verfügung

^4^Der hohe Anteil bei „Aktenlage aus sonstigen Gründen“ kann pandemiebedingt durch Telefoninterviews erklärt sein

In Bezug auf die MD-Diagnosen zeigt sich, dass Senilität (R54), Polyarthrose (M15) und nicht näher bezeichnete Demenz (F03) am häufigsten vertreten sind (Tab. 2).

**Tab. 2:** Übersicht über die 10 häufigsten erstgenannten pflegebegründende Diagnosen, *n* = 339.486

Erste pflegebegründende Diagnose			
ICD-10-Code	Bezeichnung	*n*	%
R54	Senilität	29.587	8,7
M15	Polyarthrose	22.437	6,6
F03	Nicht näher bezeichnete Demenz	19.873	5,9
R26	Störungen des Ganges und der Mobilität	19.059	5,6
I50	Herzinsuffizienz	16.468	4,9
J44	Sonstige chronische obstruktive Lungenkrankheit	10.164	3,0
U51	Kognitive Funktionseinschränkungen	9474	2,8
I51	Komplikation einer Herzkrankheit und ungenau beschriebene Herzkrankheit	8699	2,6
I63	Hirninfarkt	8637	2,5
M19	Sonstige Arthrose	7712	2,3

**Abb. 1:**
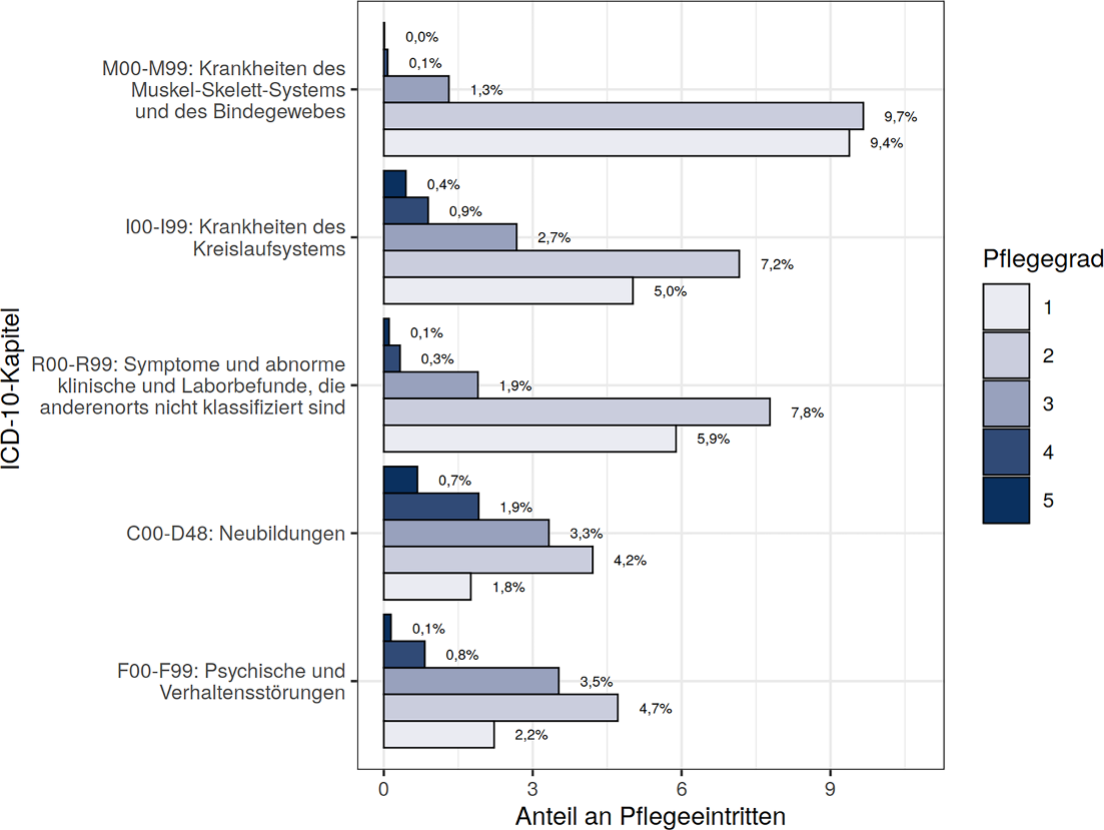
Übersicht der häufigsten erstgenannten pflegebegründenden Diagnosen, aufgeteilt nach ICD-10-Kapiteln, stratifiziert nach PG. Die %‑Angaben beziehen sich auf die Gesamtstichprobe von *n* = 339.486

Abb. 1 zeigt die 5 häufigsten ICD-10-Kapitel der MD-Diagnosen nach PG. Am häufigsten sind Krankheiten des Muskel-Skelett-Systems und des Bindegewebes (M00–M99) (20,4 %). Schwere Einschränkungen betreffen v. a. Neubildungen, Kreislaufkrankheiten und psychische Störungen, während geringe Einschränkungen meist mit Muskel-Skelett-Erkrankungen verbunden sind.

**Abb. 2:**
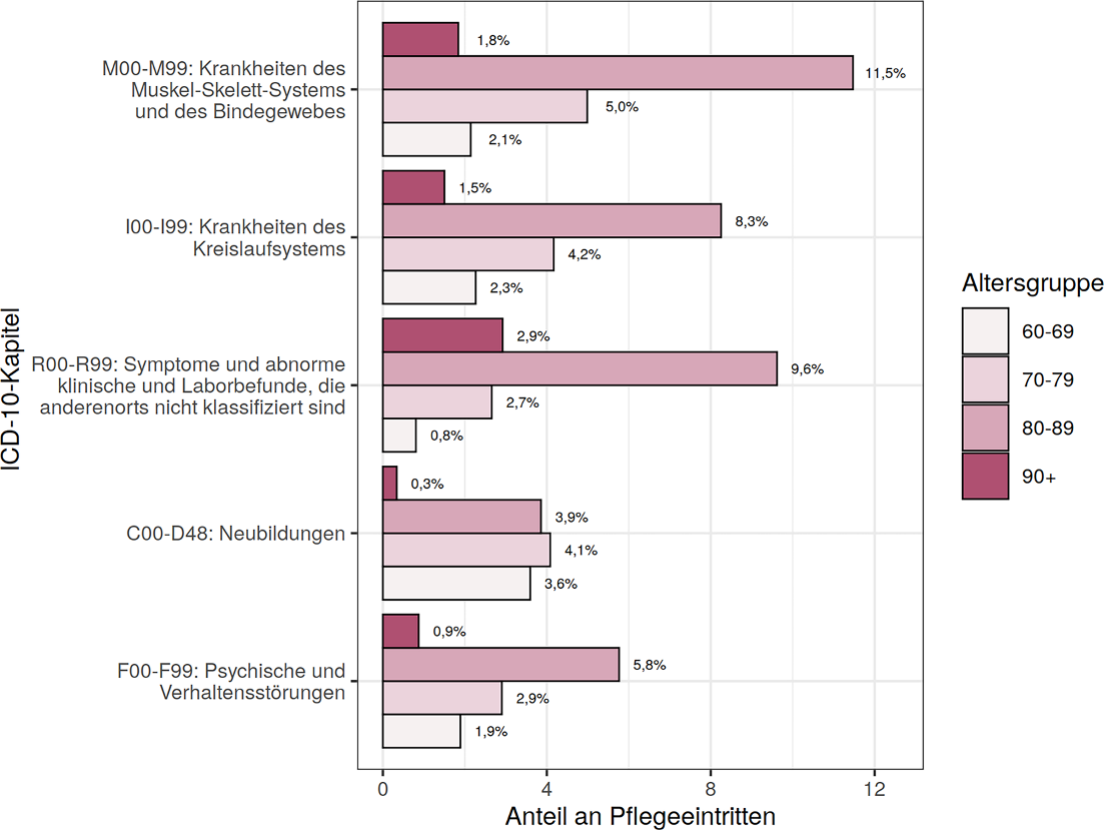
Verteilung der häufigsten erstgenannten pflegebegründenden Diagnosen nach ICD-10 Kapiteln, stratifiziert nach Altersgruppen Die %‑Angaben beziehen sich auf die Gesamtstichprobe von *n* = 339.486

**Abb. 3:**
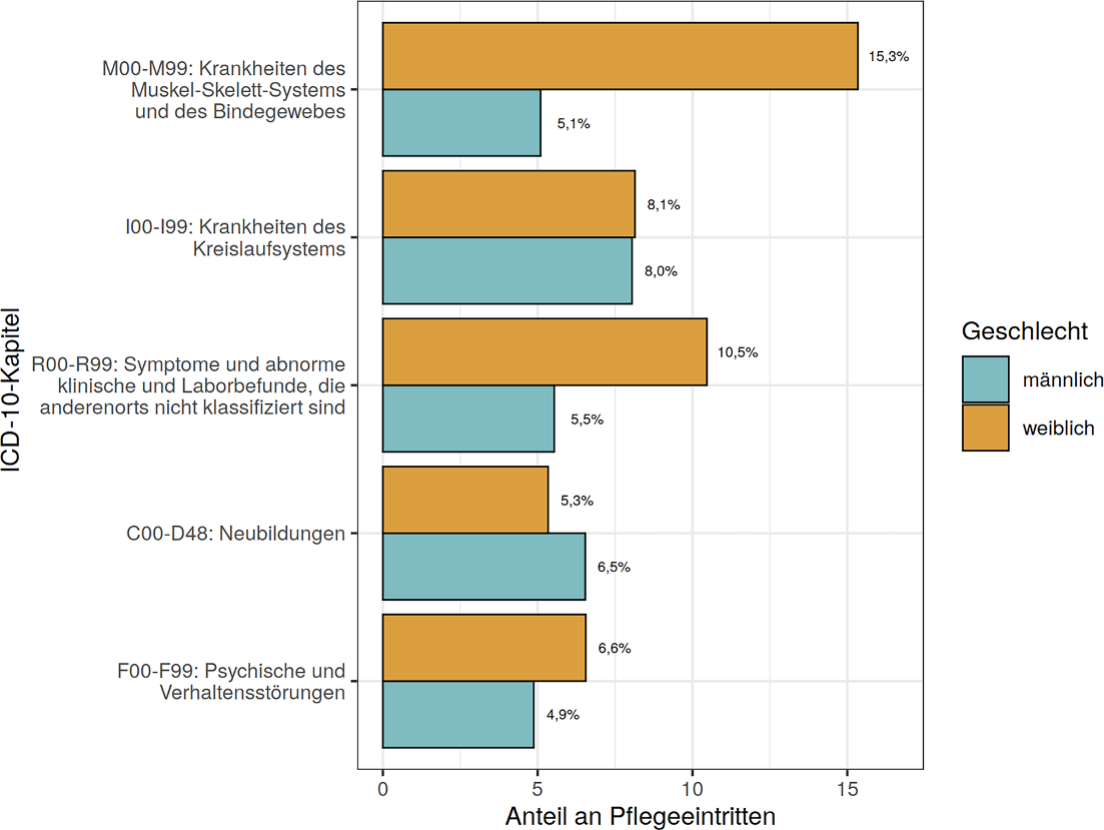
Verteilung der häufigsten erstgenannten pflegebegründenden Diagnosen nach ICD-10-Kapiteln, stratifiziert nach Geschlecht. Die %‑Angaben beziehen sich auf die Gesamtstichprobe von *n* = 339.486

Betrachtet man die MD-Diagnosen, stratifiziert nach Alter (Abb. 2) und Geschlecht (Abb. 3), so zeigen sich, dass die jüngste Altersgruppe (60 bis 69 Jahre) am häufigsten von Neubildungen betroffen ist, während bei den Ältesten (90+) die Symptomenkomplexe dominieren. Bei den Frauen überwiegen Muskel-Skelett-Erkrankungen und Symptomenkomplexe, während Männer am häufigsten von Krankheiten des Kreislaufsystems und Neubildungen betroffen sind.

**Abb. 4:**
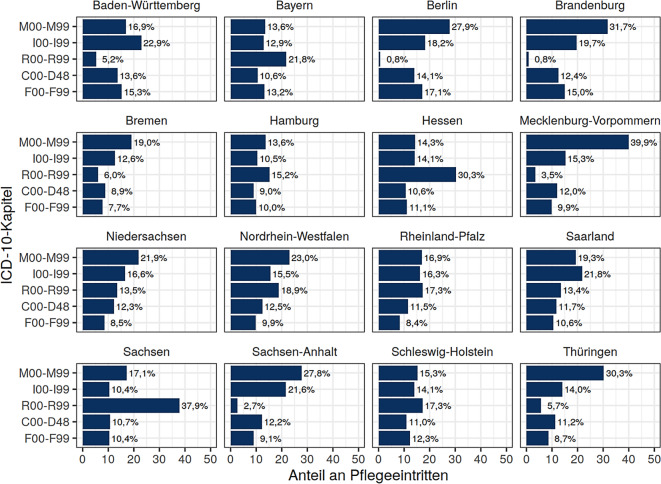
Verteilung der häufigsten erstgenannten pflegebegründenden Diagnosen nach ICD-10-Kapiteln, stratifiziert nach Bundesland. Die %‑Angaben beziehen sich auf die Stichprobe des jeweiligen Bundeslandes mit Baden-Württemberg *n* = 48.278, Bayern *n* = 49.964, Berlin *n* = 9974, Brandenburg *n* = 13.218, Bremen *n* = 2272, Hamburg *n* = 3209, Hessen *n* = 19.238, Mecklenburg-Vorpommern *n* = 9316, Niedersachsen *n* = 33.916, Nordrhein-Westfalen *n* = 57.118, Rheinland-Pfalz *n* = 15.827, Saarland *n* = 4554, Sachsen *n* = 32.769, Sachsen-Anhalt *n* = 15.651, Schleswig-Holstein *n* = 8576, Thüringen *n* = 15.606

Dabei zeigen sich auffällige regionale Unterschiede in der Codierung der MD-Diagnosen hinsichtlich Muskel-Skelett-Erkrankungen (Bayern/Hamburg 13,6 % vs. Mecklenburg-Vorpommern 39,9 %) sowie bei Symptomenkomplexen (Berlin/Brandenburg 0,8 % vs. Sachsen 37,9 %; Abb. 4).

## Diskussion

Die Befunde verdeutlichen die Charakteristika einer vulnerablen Personengruppe nach Erstantrag auf Pflegebedürftigkeit. Grundlage ist ein bundesweiter Datensatz aller AOK-Versicherten ab 60 Jahren, die 2021 erfolgreich einen Erstantrag stellten. Da die AOK knapp ein Drittel der deutschen Bevölkerung und etwa 45 % der Pflegebedürftigen versichert [12], sind ihre Datensätze besonders aussagekräftig.

Die Analyse zeigt in Bezug auf soziale Determinanten, dass Nichtalleinlebende häufiger in allen PG vertreten sind, was frühere Analysen bestätigt [9]. Andere Studien verweisen jedoch auf ein höheres Risiko von Alleinlebenden, pflegebedürftig zu sein oder zu werden [13, 14]. In dieser Analyse sind Alleinlebende v. a. in den PG 1 und 2 stärker vertreten, während in höheren PG Nichtalleinlebende dominieren, vermutlich wegen der Unterstützung durch Angehörige im selben Haushalt [15].

Die Analyse der PG-Zuweisungen zeigt, dass knapp 30 % aus den neuen Bundesländern stammen, obwohl nur etwas über 20 % der über 60-jährigen AOK-Versicherten dort wohnen [16]. Dies könnte auf gesundheitliche Ungleichheiten zwischen Ost und West zurückzuführen sein. Die Lebenserwartung der Männer im Osten ist niedriger [17], die Versorgungssituation in ländlichen Gebieten schlechter und die subjektive Gesundheit wird schlechter bewertet [18]. Zudem ist der Altersdurchschnitt der Bevölkerung im Osten höher [19] , und Prognosen erwarten dort den größten Zuwachs an Pflegebedürftigen, teils bedingt durch den Wegzug der jüngeren Generation nach der Wiedervereinigung [20], teils bedingt durch den Wegzug der jüngeren Generation – der potenziell Pflegenden – nach der Wiedervereinigung.

In dieser Analyse wurde in 61,2 % der Fälle die Begutachtung als „Aktenlage aus sonstigen Gründen“ durchgeführt, vermutlich aufgrund pandemiebedingter Umstellungen auf telefonische Interviews. Auch 2022 wurden noch in über 30 % der Fälle telefonische Begutachtungen durchgeführt, um besonders vulnerable Zielgruppen zu schützen [21]. Bei den pflegebegründenden Diagnosen dominieren 5 Hauptgruppen nach ICD-10: Muskel-Skelett-Erkrankungen, Krankheiten des Kreislaufsystems, Symptome und abnorme klinische Befunde, Neubildungen sowie psychische und Verhaltensstörungen. Besonders auffällig ist, dass Neubildungen bei jüngeren Altersgruppen höher sind, mehr Männer betreffen und häufiger mit höheren PG (3–5) einhergehen. Muskel-Skelett-Erkrankungen, die zu geringeren Einschränkungen führen und in niedrigere PG eingestuft werden, sind häufiger bei Frauen vertreten. Unterschiede zwischen den Bundesländern sind besonders bei Muskel-Skelett-Erkrankungen und Symptomen erkennbar, mit Abweichungen von bis zu 36 %. Ein Teil dieser Unterschiede könnte auf die Begutachtungspraxis zurückzuführen sein. Berlin und Brandenburg, für die ein gemeinsamer MD zuständig ist, zeigen ähnliche Diagnosemuster, während die anderen 14 Bundesländer mit eigenen MD unterschiedliche Profile aufweisen. Die Ergebnisse von Schütz et al. [8] und Braseke et al. [6] unterstützen diese Befunde und zeigen, dass die Unterschiede in der Codierung der pflegebegründenden Diagnosen weiter untersucht werden sollten, um ihre Aussagekraft zu bewerten.

### Limitationen

Neben den Stärken vorliegender Studie (hohe Fallzahl, deutschlandweite Untersuchung, bisher wenig analysierte Daten) sind folgende Limitationen zu benennen:

- Aussagen beziehen sich nur auf Erstantragstellende, denen ein PG zugesprochen wurde.
- Durch die pandemiebedingte Umstellung auf strukturierte Telefoninterviews können Verzerrungen auf das Begutachtungsergebnis nicht ausgeschlossen werden.
- Pflegebegutachtung als Momentaufnahme, die möglicherweise nicht das umfassende Bild einer Versorgungsituation abdecken kann.
- MD-Daten liefern nur wenige soziodemografische Angaben.
- Einige Personengruppen möglicherweise unterrepräsentiert aufgrund sozialer Ungleichheit im Antragsverhalten oder durch Selektionseffekte, wenn pandemiebedingt Begutachtungen verschoben oder nicht wahrgenommen wurden.
- Das Merkmal „pflegebegründende Diagnose“ wird nicht qualitätsgesichert erhoben.

### Ausblick

Die Charakteristika von Erstantragsstellenden auf Leistungen aus der Pflegeversicherung können bedeutende Anhaltspunkte für die zukünftige Forschung zu Präventionsmöglichkeiten von Pflegebedürftigkeit liefern. Dies sollte eine Zukunftsaufgabe für alle relevanten Stakeholder in der Gesundheitslandschaft – insbesondere auch für die Krankenkassen – sein, da Prävention, gerade auch bei Pflegebedürftigkeit, nach wie vor ein Desiderat in Deutschland darstellt. Der große Anteil von Ersteinstufungen in niedrige PG weist darauf hin, dass insbesondere zielgruppenspezifische und geschlechterspezifische Präventionsansätze, die den Grad der Selbstständigkeit erhalten oder die Progredienz abmildern, sinnvoll sind. Darüber hinaus sollte verstärkt die Wohnsituation (z. B. alleinlebend) berücksichtigt werden, um inner- und außerfamiliäre Unterstützungspotenziale miteinbeziehen zu können. Besonders für alleinlebende Personen erscheinen wohnortnahe Strukturen oder Ansätze wie etwa der des Community Health Nursing als bedeutende Ressourcen.

Die vorliegende Analyse zeigt regionale Differenzen, sowohl zwischen Ost und West als auch bei der Codierung der MD-Diagnosen, was in weiteren Studien untersucht werden sollte. Zudem ist es essenziell, die Entwicklung der Pflegebedürftigkeitsquote weiterzubeobachten, da diese die demografischen Erwartungen übertrifft und nur teilweise durch den Einführungseffekt der Pflegereform von 2017 oder Nachholeffekte durch die Coronapandemie erklärt werden kann. Langzeitstudien sind hierbei unerlässlich, um die langfristigen Trends und Einflussfaktoren der Pflegebedürftigkeit tiefgreifend zu verstehen und effektive Präventionsstrategien zu entwickeln.

## Fazit für die Praxis

- Drei Viertel der erstmalig Pflegebedürftigen wurden in niedrige Pflegegrade (PG) eingestuft. Insbesondere für diese – noch über Selbstständigkeitspotenziale verfügende – Gruppe sind präventive und rehabilitative Interventionen in hohem Maße indiziert, um eine weitere Progredienz zu vermeiden. Dabei sollte die Gruppe der Alleinlebenden besondere Beachtung finden, und wohnortnahe Strukturen sollten gestärkt werden.
- Bei den MD (Medizinischer Dienst)-Diagnosen überwogen Senilität (R54), Polyarthrose (M15) und Demenz (F03), welche in den Fokus der Prävention gesetzt werden sollen.
- Die Pflegebegutachtung sollte weiterentwickelt werden, insbesondere im Hinblick auf Sicherstellung der Konsistenz und Qualität der Dokumentation pflegebegründender Diagnosen.

## Data Availability

Die Daten sind nicht allgemein verfügbar.
